# Associations between lead concentrations and cardiovascular risk factors in U.S. adolescents

**DOI:** 10.1038/s41598-017-09701-4

**Published:** 2017-08-22

**Authors:** Cheng Xu, Yaqin Shu, Zhi Fu, Yuanli Hu, Xuming Mo

**Affiliations:** 1 Department of Cardiothoracic Surgery, Children’s Hospital of Nanjing Medical University, Nanjing, 210008 China; 2 Department of Thoracic Surgery, Huai’an First People’s Hospital, Nanjing Medical University, Huai’an, 223001 China

## Abstract

Little is known regarding the effects of environmental lead exposure on cardiovascular risk factors in the adolescent population. We studied 11,662 subjects included in the National Health and Nutrition Examination Survey (NHANES) 1999–2012. Blood lead levels were analysed for their association with cardiovascular risk factors (CVRF). Regression coefficients (Beta) and 95% confidence intervals (CIs) of blood lead in association with CVRF (e.g., total cholesterol, HDL-cholesterol, LDL-cholesterol, triglyceride, fasting glucose, glycohemoglobin, fasting insulin, and blood pressure) were estimated using multivariate and generalized linear regression after adjusting for age, gender, ethnicity, serum cotinine, body mass index (BMI), physical activity, and household income. We identified a strong positive association between blood lead (coefficient = 0.022, 95% CI 0.003, 0.041; P = 0.022) and LDL-cholesterol in adolescents (age 12–19 years). However, no associations with other CVRFs were found in the overall population. In the generalized linear models, participants with the highest lead levels demonstrated a 1.87% (95% CI 0.73%, 3.02%) greater increase in serum LDL-cholesterol (p for trend = 0.031) when compared to participants with the lowest lead levels. These results provide epidemiological evidence that low levels of blood lead are positively associated with LDL-cholesterol in the adolescent population.

## Introduction

Cardiovascular disease (CVD) has become the leading cause of death in the world over the past two decades, regardless of economic status. It also has caught up with infectious diseases as the leading cause of death, and its impact threatens national economies and households^1, 2^. Recently, several research studies have showed CVD presents a significant public health concern in adolescents^3, 4^. Cardiovascular risk factors (CVRF), such as blood pressure, fasting glucose and insulin, glycohaemoglobin (HbA1c) and lipid profiles, were sensitive markers to evaluate CVD occurrence in populations. Reportedly, genetic factors, environmental factors and behavioural habits could affect CVRF in adolescents^5–7^. An increasing attention on the associations between low concentration environmental chemicals and CVRF in adolescents has been raised. Environmental exposure is a crucial but underappreciated risk factor that contributes to the progress and severity of CVD.

Lead, a heavy metal, is known as one of the most common environmental toxins, resulting in neuropsychological and functional decline in humans^8^. According to the U.S. Centers for Disease Control and Prevention (CDC), at least 4 million households have adolescents that have been exposed to high levels of lead. Currently no definite level of blood lead is considered safe and the CDC has established 100 µg/L lead as the level of concern. In recent years, blood lead levels among adolescents have been investigated worldwide. In China, median levels of blood lead levels in the male and female population aged 0–18 years old were 48.8 μg/L and 46.1 μg/L^9^, respectively. In the United States, the average blood lead concentrations were 22.8 μg/L, 45.5 μg/L, and 40.7 μg/L for Caucasian, black, and Hispanic and Mexican American adolescents, respectively^10^. Low lead concentrations may impair human health apart from neurotoxicity, and even exposure below current regulatory standards can lead to adverse effects on cardiovascular health. However, no data exist to date regarding low lead levels and CVRF in adolescents.

In the present study, nationally representative data from the National Health and Nutrition Examination Survey (NHANES) 1999–2012 was analysed to explore the associations between lead exposure biomarkers and CVRF (blood pressure, fasting glucose and insulin, HbA1c and lipid profiles) in adolescents (12 to 19-year-olds).

## Results

**Figure 1:**
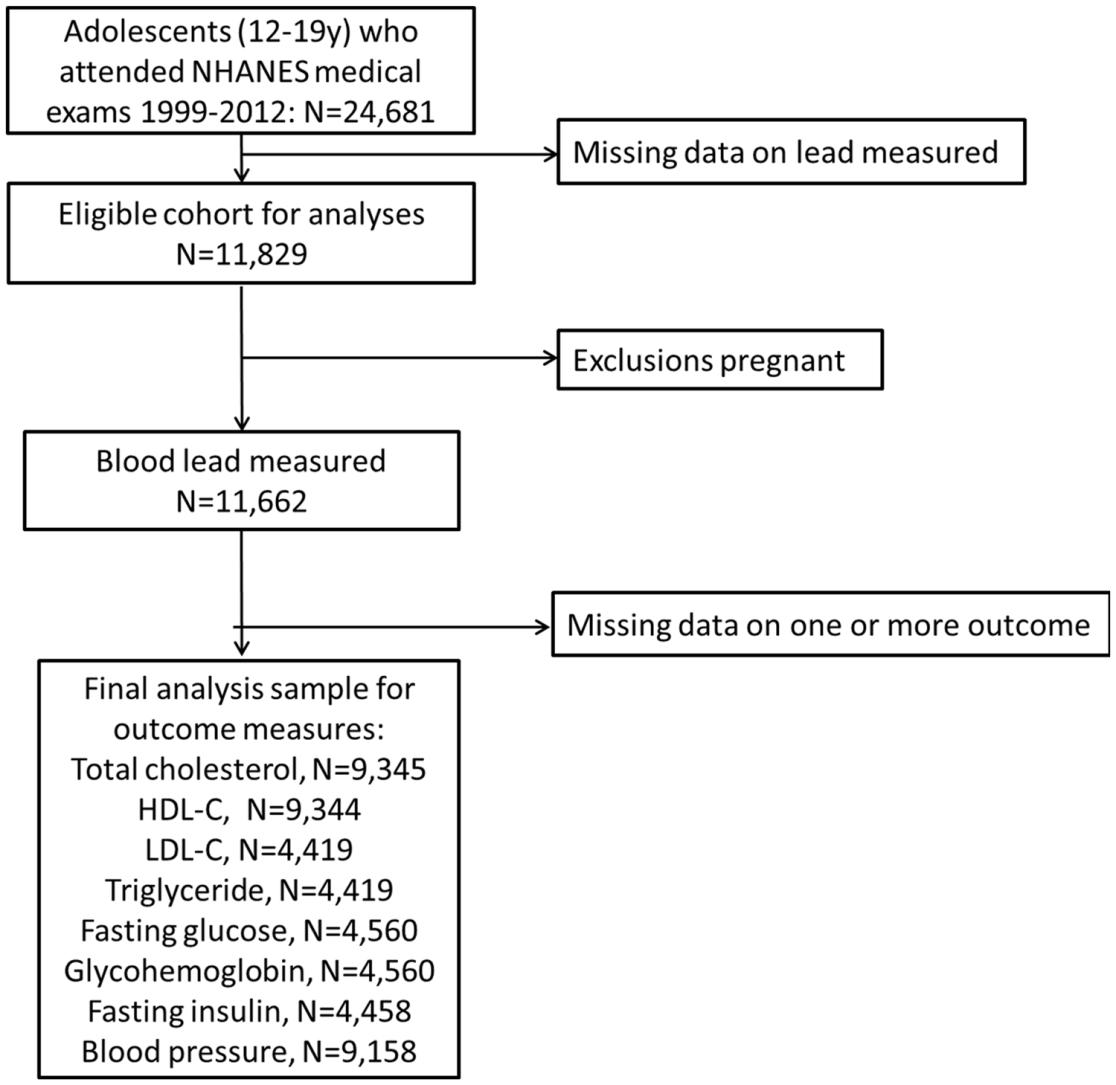
Eligible participants and those included in the analyses of the associations between blood lead exposure and CVD risk factors in adolescents.

Of the 71,916 participants of the NHANES 1999–2012, we excluded those who were aged age greater than 20 years old (n 38,024) and less than 12 years old (n 9,211), who had missing blood lead (n 12,852), or who were pregnant (n 167). The final analytic population included 11,662 participants (6,031 boys and 5,631 girls; Fig. 1).

Table 1 shows the mean ± standard deviation was 1.17 ± 1.20 μg/dL for blood lead. Blood lead concentrations were significantly higher in participants who were younger, were boys, were Mexican American, had lower income, had a lower body mass index, had lower waist circumference, had high physical activity or had high serum cotinine. Blood lead concentrations also differed by cycles of NHANES.

**Table 1:** Blood lead concentration (mean ± SD) for participants according to demographic.

Characteristics	Participants (n)	Blood lead (μg/dL)	P value
Overall	11,662	1.17 ± 1.20	
Age (years)			<0.001
12–15	5,872	1.20 ± 1.03	
16–19	5,790	1.14 ± 1.35	
Gender			<0.001
Boy	6,031	1.39 ± 1.43	
Girl	5,631	0.94 ± 0.82	
Ethnicity			<0.001
Mexican American	3,668	1.29 ± 1.26^a^	
Other Hispanic	734	1.04 ± 0.86	
Non-Hispanic White	3,074	0.97 ± 1.01	
Non-Hispanic Black	3,518	1.28 ± 1.36	
Other Race - Including Multi-Racial	668	1.01 ± 0.80	
PIR			<0.001
<1	3,533	1.39 ± 1.54	
≥1	7,259	1.05 ± 0.97	
Serum cotinine (ng/mL)			<0.001
<LOD (0.011)	1,720	0.83 ± 0.82	
LOD-10	8,278	1.21 ± 1.25	
≥10	1,455	1.34 ± 1.24^a^	
Waist circumference (cm)^b^			<0.001
Tertile 1	3,813	1.26 ± 1.10^a^	
Tertile 2	3,826	1.18 ± 1.47	
Tertile 3	3,781	1.08 ± 0.97	
BMI (kg/m^2^)^c^			<0.001
Tertile 1	3,854	1.27 ± 1.53^a^	
Tertile 2	3,843	1.17 ± 1.01	
Tertile 3	3,839	1.08 ± 0.95	
Television, video game and computer usage (hours)			<0.001
≤2	2,834	1.21 ± 1.12	
>2	4,221	1.17 ± 1.42	
NHANES cycles			<0.001
1999–2000	2,111	1.52 ± 1.26^a^	
2001–2002	2,185	1.28 ± 1.15	
2003–2004	2,029	1.30 ± 1.23	
2005–2006	1,951	1.12 ± 1.64	
2007–2008	1,074	0.99 ± 0.73	
2009–2010	1,183	0.84 ± 0.68	
2011–2012	1,129	0.68 ± 0.55	

^a^Significant higher than other group by post hoc multiple comparisons.

^b^Mean values ± SD: 81.7 ± 14.9 cm. Tertile ranges (cm): tertile 1, 47.1–73.3; tertile 2, 73.4–84.4; tertile 3, 84.5–179.0.

^c^Mean values ± SD: 24.0 ± 6.0 kg/m^2^. Tertile ranges (kg/m^2^): tertile 1, 13.1–20.7; tertile 2, 20.8–24.9; tertile 3, 25.0–66.3.

PIR: poverty income ratio, LOD: limit of detection.

**Figure 2:**
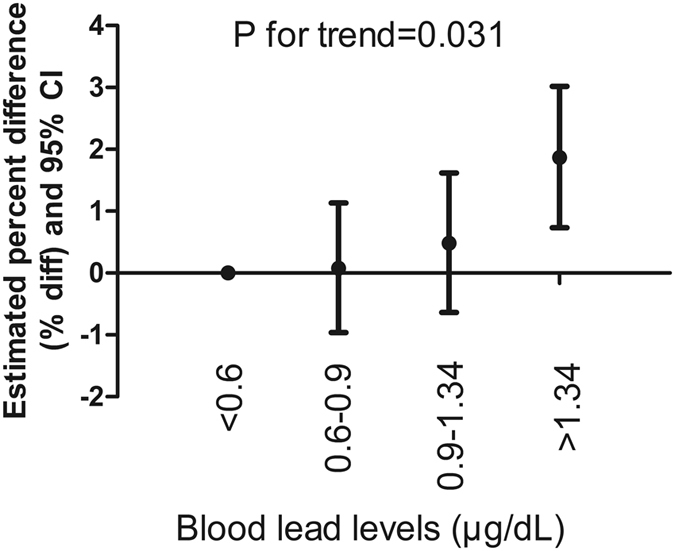
Estimated percent difference (% diff) and 95% Confidence Intervals (95% CI) in serum LDL-C concentrations in U.S. adolescents 1999–2012 for each interquartile ratio (IQ Ratio) increase in blood lead levels.

**Figure 3:**
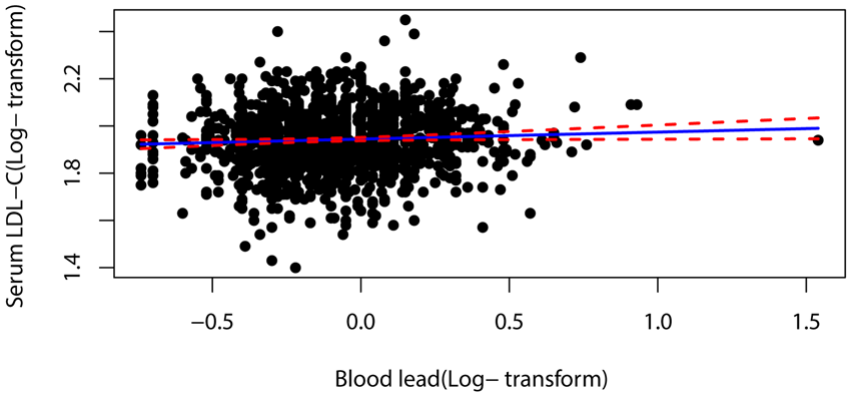
A scatter plot and a fitted line with 95% CI of the relationship between blood lead and LDL-C in adolescents.

Table S1 shows mean, median, and geometric mean of CVRF. Table 2 shows multivariable associations of blood lead with CVRF in U.S. adolescents 1999–2012. In age and gender-adjusted models, blood lead was positively associated with HDL-C (coefficient = 0.008, 95% CI: 0.000, 0.016), but the association moved substantially towards the null after adjustment for other covariates (coefficient = 0.003, 95% CI: −0.005, 0.011). Blood lead levels were inversely associated with fasting glucose (coefficient = −0.005, 95% CI: −0.007, −0.002), HbA1c (coefficient = −0.006, 95% CI: −0.008, −0.003), and fasting insulin (coefficient = −0.032, 95% CI: −0.047, −0.017) in age and gender-adjusted models. However, the association moved substantially towards the null in the fully adjusted model. Blood lead was positively associated with LDL-C in age-adjusted, gender-adjusted, and fully adjusted models. Table 3 suggests blood lead concentrations in the highest quartile compared with the lowest quartile were associated with higher LDL-C (coefficient = 0.023, 95% CI: 0.009, 0.037), with evidence of a dose-response relationship (P for trend = 0.031). Similarly, subjects in the highest lead quintile had mean LDL-C levels that were 1.87% greater (95% CI: 0.73%, 3.02%) than in the lowest quintile (Fig. 2). The results did not meaningfully differ after replacing BMI with waist circumference. The relationship between blood lead and LDL-C in adolescents is visualized in a scatter plot and a fitted line with 95% CI (Fig. 3).

**Table 2:** Multivariable associations of serum lead with cardiovascular risk factors in US adolescents 1999–2012.

CVD risk factors	N	Model 1			Model 2			Model 3		
		Coefficient	95% CI	P Value	Coefficient	95% CI	P Value	Coefficient	95% CI	P Value
Total cholesterol	9,345	0.004	−0.002 to 0.010	0.190	0.005	−0.001 to 0.012	0.088	0.006	−0.001 to 0.013	0.077
HDL-C	9,344	0.008	0.000 to 0.016	0.038	0.010	0.002 to 0.018	0.012	0.003	−0.005 to 0.011	0.452
LDL-C	4,419	0.019	0.006 to 0.032	0.005	0.024	0.010 to 0.038	0.001	**0**.**022**	**0**.**003 to 0**.**041**	**0**.**022**
Fasting triglyceride	4,419	−0.010	−0.021 to 0.002	0.093	−0.013	−0.025 to −0.002	0.021	−0.011	−0.024 to 0.001	0.605
Fasting glucose	4,560	−0.005	−0.007 to −0.002	0.001	−0.005	−0.008 to −0.002	<0.001	0.001	−0.002 to 0.004	0.339
Glycohaemoglobin	4,560	−0.006	−0.008 to −0.003	<0.001	−0.006	−0.009 to −0.004	<0.001	0.003	0.000 to 0.005	0.069
Fasting insulin	4,458	−0.032	−0.047 to −0.017	<0.001	−0.039	−0.054 to −0.024	<0.001	0.008	−0.007 to 0.024	0.308
Systolic blood pressure	9,201	−0.003	−0.006 to 0.000	0.046	−0.003	−0.006 to 0.000	0.072	−0.001	−0.004 to 0.003	0.117
Diastolic blood pressure	9,158	0.005	−0.002 to 0.011	0.179	0.006	−0.001 to 0.013	0.077	0.000	−0.009 to 0.009	0.960

Model 1—age and gender adjusted; model 2—as model 1 plus ethnicity, PIR; model 3—as model 2 plus waist circumference, serum cotinine, physical activity and NHANES cycles.

CVD, cardiovascular disease; CI, confidence interval; HDL-C, high-density lipoprotein cholesterol; LDL-C, low-density lipoprotein cholesterol; PIR, poverty index ratio.

**Table 3:** Estimated coefficient (beta) and 95% Confidence Intervals (95% CI) in CVD risk factors in US adolescents 1999–2012 for each quartile increase in blood lead levels.

CVD risk factors	Quartile 1	Quartile 2	Quartile 3	Quartile 4	P for trend
Total cholesterol	Reference	−0.002 (−0.007, 0.002)	0.001 (−0.004, 0.005)	−0.001 (−0.006, 0.004)	0.046
HDL-C	Reference	0.002 (−0.004, 0.008)	0.004 (−0.002, 0.010)	−0.003 (−0.009, 0.003)	0.939
LDL-C	Reference	0.001 (−0.012, 0.014)	0.006 (−0.008, 0.020)	**0**.**023 (0**.**009**, **0**.**037)**	0.031
Fasting triglyceride	Reference	−0.018 (−0.036, −0.001)	−0.005 (−0.023, 0.012)	−0.013 (−0.031, 0.005)	0.452
Fasting glucose	Reference	−0.005 (−0.010, 0.000)	−0.004 (−0.009, 0.002)	−0.012 (−0.017, −0.007)	0.327
Glycohaemoglobin	Reference	−0.003 (−0.005, 0.000)	−0.003 (−0.005, 0.001)	−0.002 (−0.005, −0.000)	0.086
Fasting insulin	Reference	0.007 (−0.014, 0.027)	0.002 (−0.019, 0.023)	−0.006 (−0.027, 0.015)	0.270
Systolic blood pressure	Reference	0.002 (0.000, 0.004)	0.002 (0.000, 0.004)	0.001 (−0.001, 0.004)	0.434
Diastolic blood pressure	Reference	0.000 (−0.007, 0.006)	0.002 (−0.005, 0.009)	0.001 (−0.006, 0.008)	0.406

Model was adjusted as age, gender, ethnicity, PIR, BMI, serum cotinine, and physical activity.

CVD, cardiovascular disease; HDL-C, high-density lipoprotein cholesterol; LDL-C, low-density lipoprotein cholesterol; PIR, poverty index ratio.

Lead(μg/dL), Quartile 1: <0.6; Quartile 2: 0.6–0.9; Quartile 3: 0.9–1.34; Quartile 4: >1.34.

We take into account that blood lead concentrations were higher in participants who were younger, were boys, were Mexican American, had lower income, had a lower body mass index, had lower waist circumference, had high physical activity or had high serum cotinine. Due to these results, we further divided the population into subgroups to examine the correlations. Table 4 shows that in participants who were younger, were boys, were Non-Hispanic White, had higher income, had a lower body mass index, had lower waist circumference, had low physical activity and had medium serum cotinine were significant of blood lead highest quartile compared with the lowest quartile were associated with higher LDL-C.

**Table 4:** Estimated coefficient (beta) and 95% Confidence Intervals (95% CI) in serum low-density lipoprotein cholesterol in US adolescents 1999–2012 for each quartile increase in blood lead levels stratification by different covariates

LDL-C	Quartile 2	Quartile 3	Quartile 4	P for trend
**Age (years)**				
12–15	0.013 (−0.005, 0.031)	0.017 (−0.001, 0.036)	**0**.**028 (0**.**009**, **0**.**048)**	<0.001
16–19	−0.007 (−0.025, 0.011)	−0.003 (−0.023, 0.016)	0.012 (−0.008, 0.032)	0.133
**Gender**				
Boy	0.016 (−0.005, 0.037)	0.016 (−0.005, 0.037)	**0**.**033 (0**.**012**, **0**.**053)**	0.001
Girl	−0.002 (−0.017, 0.014)	0.012 (−0.006, 0.031)	0.016 (−0.004, 0.037)	0.008
**Ethnicity**				
Mexican American	−0.004 (−0.025, 0.018)	0.015 (−0.007, 0.038)	0.021 (−0.001, 0.043)	0.001
Other Hispanic	−0.011 (−0.060, 0.038)	−0.046 (−0.113, 0.022)	0.009 (−0.048, 0.065)	0.622
Non-Hispanic White	0.019 (−0.004, 0.042)	0.031 (0.004, 0.057)	**0**.**047 (0**.**017**, **0**.**076)**	0.009
Non-Hispanic Black	−0.001 (−0.027, 0.025)	−0.010 (−0.037, 0.016)	0.007 (−0.20, 0.033)	0.342
Other Race - Including Multi-Racial	0.001 (−0.048, 0.049)	0.045 (−0.010, 0.099)	0.031 (−0.029, 0.091)	0.135
**PIR**				
<1	0.007 (−0.018, 0.031)	0.020 (−0.005, 0.044)	0.009 (−0.015, 0.033)	0.023
≥1	0.004 (−0.011, 0.018)	0.005 (−0.011, 0.022)	**0**.**035 (0**.**018**, **0**.**052)**	<0.001
**Serum cotinine (ng/mL)**				
<LOD (0.011)	0.019 (−0.007, 0.045)	0.015 (−0.015, 0.045)	0.037 (0.001, 0.073)	0.012
LOD-10	0.004 (−0.011, 0.019)	0.006 (−0.010, 0.022)	**0**.**017 (0**.**001**, **0**.**033)**	0.007
≥10	−0.023 (−0.068, 0.022)	0.010 (−0.037, 0.057)	0.022 (−0.024, 0.068)	0.082
**Waist circumference (cm)** ^**b**^				
Tertile 1	0.006 (−0.017, 0.029)	0.009 (−0.015, 0.034)	**0**.**029 (0**.**004**, **0**.**053)**	0.010
Tertile 2	−0.005 (−0.026, 0.016)	0.013 (−0.009, 0.036)	0.022 (−0.001, 0.045)	0.015
Tertile 3	0.010 (−0.012, 0.031)	0.005 (−0.018, 0.028)	0.012 (−0.012, 0.037)	0.180
**BMI (kg/m** ^**2**^ **)** ^**c**^				
Tertile 1	0.009 (−0.013, 0.032)	0.013 (−0.011, 0.037)	**0**.**027 (0**.**003**, **0**.**051)**	0.003
Tertile 2	−0.001 (−0.023, 0.021)	0.009 (−0.014, 0.033)	0.020 (−0.005, 0.044)	0.058
Tertile 3	0.001 (−0.020, 0.022)	0.002 (−0.021, 0.025)	0.016 (−0.008, 0.039)	0.121
**Television**, **video game and computer usage (hours)**				
≤2	0.002 (−0.018, 0.022)	0.007 (−0.015, 0.028)	0.014 (−0.008, 0.035)	0.039
>2	0.006 (−0.010, 0.022)	0.013 (−0.005, 0.030)	**0**.**030 (0**.**011**, **0**.**048)**	<0.001
**NHANES cycles**				
1999–2000	−0.005 (−0.045, 0.036)	0.001 (−0.041, 0.043)	0.022 (−0.019, 0.062)	0.080
2001–2002	0.019 (−0.014, 0.051)	0.029 (−0.005, 0.063)	0.031 (−0.004, 0.06)	0.160
2003–2004	−0.008 (−0.035, 0.018)	−0.014 (−0.041, 0.013)	0.005 (−0.022, 0.033)	0.294
2005–2006	0.004 (−0.019, 0.027)	−0.002 (−0.027, 0.024)	−0.001 (−0.028, 0.025)	0.894
2007–2008	0.012 (−0.018, 0.043)	0.021 (−0.013, 0.054)	0.037 (−0.002, 0.077)	0.330
2009–2010	0.008 (−0.018, 0.033)	0.013 (−0.017, 0.043)	0.049 (0.009, 0.089)	0.058
2011–2012	−0.006 (−0.036, 0.023)	0.042 (0.004, 0.080)	0.018 (−0.038, 0.073)	0.033

Model was adjusted as age, gender, ethnicity, PIR, waist circumference, serum cotinine, and physical activity.

CVD, cardiovascular disease; HDL-C, high-density lipoprotein cholesterol; LDL-C, low-density lipoprotein cholesterol; PIR, poverty index ratio.

Lead(μg/dL), Quartile 1: <0.6; Quartile 2: 0.6–0.9; Quartile 3: 0.9–1.34; Quartile 4: >1.34.

^b^Mean values ± SD: 81.7 ± 14.9 cm. Tertile ranges (cm): tertile 1, 47.1–73.3; tertile 2, 73.4–84.4; tertile 3, 84.5–179.0.

^c^Mean values ± SD: 24.0 ± 6.0 kg/m^2^. Tertile ranges (kg/m^2^): tertile 1, 13.1–20.7; tertile 2, 20.8–24.9; tertile 3, 25.0–66.3.

## Discussion

Using data from a large population sample in the U.S. NHANES 1999–2012 survey, we found that blood lead levels were positively associated with serum LDL-C levels in adolescents. This association persisted after adjusting for age, gender, ethnicity, PIR, BMI, serum cotinine, and physical activity. Further subgroup analyses showed that the associations between blood lead levels and LDL-C concentrations remained significant in subjects who were 12–15 years old, boys, and Non-Hispanic White, with low physical activity, low waist circumference and low BMI in our study.

On the epidemiological level, previous studies revealed relationships between human blood lead levels and both CVD mortality and CVRF. Aoki *et al*. found blood lead was linearly associated with increased CVD mortality in the U.S. population aged 40 years and older^11^. With lead occupational exposure, such as with bus drivers, it was discovered that blood lead level may contribute to the risk of the CVD^12^. A total of 7,383 South Korean lead workers demonstrated that increasing blood lead levels was correlated with an increased risk of high blood pressure^13^. Additionally, Prokopowicz *et al*. revealed that, with 231 male adults, non-occupational exposure to lead was correlated with new risk factors for CVD, in detail L-homoarginine, fibrinogen, C-reactive protein and homocysteine^14^. For CVRF, Peters *et al*. observed that blood lead levels were positively associated with total cholesterol and HDL levels among 426 elders^15^. Rhee *et al*. discovered positive associations between blood lead levels and metabolic syndrome^16^. In addition, an occupational exposure study by Ghiasvand *et al*. showed that there was no significant correlation between blood lead levels and LDL-C in 497 male workers^17^, which is not consistent with our results. The explanation may be that the blood lead concentration and ages were effect factors.

The roles of lead in the hyperlipidaemia in animal models has been reported. Newairy *et al*. used female rats treated with tape water containing 0 and 200 mg/L lead acetate for three weeks to evaluate lipid profile^18^. Total lipids, cholesterol, triglycerides and LDL-C were significantly increased and HDL-C was decreased in lead acetate exposed rats compared to the control group. Similarly, Abdel-Moneim *et al*. treated male rats with 25 mg of lead acetate/kg body weight once daily for 7 day by intraperitoneal injection, indicating that cholesterol, triglycerides and LDL-C were elevated and HDL-C was reduced compared to the control group^19^. Additionally, Komousani *et al*. administered lead acetate (100 mg/kg body weight) for 2 weeks by subcutaneous in male rats, suggesting high levels of LDL-C and a sharp drop in HDL-C compared to the control group^20^. Although the dose and exposure methods used in the rat models were various, this evidence may support our findings about positive associations between blood lead levels and serum LDL-C levels in adolescents.

In our study, participants who were 12–15 years old, boys, and Non-Hispanic White, and who had low physical activity, low waist circumference and low BMI exhibited significant associations of blood lead and serum LDL-C. The reasons that these factors presented different results were unknown. We speculated that young populations are more likely to put things containing lead into their mouths. An increasing amount of evidence suggests that malnutrition is highly significantly associated with increased levels of blood lead^21^, which may explain why the lower waist circumference and BMI subjects generated positive results.

Non-occupational lead exposure may result in adverse health, even in adolescents, in our findings. Keeping lead and lead sources out of the reach of children and adolescents should be a priority among priorities. Many lead sources were listed: workplace, lead-based paint, leaded gasoline, water, soil, and food contaminated with lead. As for children and adolescents, the sources of lead were as follows: (a) in nonresidential settings and older homes lead paint was used before being banned; (b) leaded gasoline was used in developing countries; (c) lead-leaden kitchenware are sources of lead contamination in food, and old public water systems continue to have networks that include lead piping. We should recognize that lead exposure is wide, and that the adverse effects of lead may contribute to poor human health.

Our results were the first to report the positive associations between blood lead levels and serum LDL-C in U.S. adolescents. The advantages were as follows: (a) the blood lead was due to non-occupational exposure; (b) a large population was included in our present study; and (c) concomitant variables were available to adjust our analyses model. Notably, there are several limitations to the present study. First, due to the cross-sectional character of our study, we were unable to determine whether lead affected LDL-C or vice versa. Second, genetic predisposition may be a crucial influencing factor that was not measured in the NHANES. Third, we could not determine the exposure routes of lead to provide preventive measures to the government.

In summary, the present study results revealed that lead exposure may be associated with harmful CVRF in U.S. adolescents. Future studies are needed to validate this and explore potential mechanisms.

## Methods

### Study population

The NHANES studies were performed by the US National Center for Health Statistics (Centers for Disease Control and Prevention, Atlanta, GA, USA). All cycles of the NHANES protocols were approved by the NCHS Research Ethics Review Board^22^, and data user agreement was online (https://www.cdc.gov/nchs/data_access/restrictions.htm). It is a cross-sectional study with a nationally representative sample of the civilian, non-institutionalized U.S. population. The subjects enrolled in the present study were obtained from seven cycles of the NHANES (1999–2000, 2001–2, 2003–4, 2005–6, 2007–8, 2009–10 and 2011–2). This cross-sectional dataset is comprised of health questionnaire, laboratory (i.e. blood lead, total cholesterol, HDL-cholesterol, LDL-cholesterol, triglyceride, fasting glucose, glycohemoglobin, fasting insulin, and serum cotinine). Interviews, substantial physical examinations, and laboratory testing (including blood collection) were conducted in the Mobile Examination Centers. All data from the website of the National Center for Health Statistics were retrieved^23^. We examined the associations of blood lead with CVRF (blood pressure, fasting glucose and insulin, HbA1c and lipid profiles) in adolescents who participated in the NHANES.

Subjects in the NHANES over a range of 14 years were chosen to form a random subgroup for the detection of blood lead and CVRF. There were two primary exclusion criteria of subjects, including an age greater than 20 years and a lack of lead measurement. Pregnant women were excluded from the study since they are considered to have an abnormal physiological status that prevents the accuracy of the CVRF.

The final investigation sample consisted of 11,662 adolescents (6–19 years of age, 6,031 boys and 5,631 girls) from this subgroup.

### Blood lead and cardiovascular risk factors detection

The measurements of lead concentrations were described in detail elsewhere^24^. Cardiovascular risk factors were determined from serum and physical examination. Total cholesterol, triglycerides, HDL-C and LDL-C^25^, glucose^26^, HbA1c^27^, and insulin^28^ were detected in serum. All BP determinations (systolic and diastolic) were averaged in further statistical analysis.

### Covariates

We controlled for the following a priori confounders of the relation between blood lead levels and CVRF: age, gender, ethnicity, serum cotinine, the poverty income ratio (PIR), body mass index (BMI), waist circumference, and physical activity. Serum cotinine, a marker of exposure to environmental tobacco smoke, was categorized as less than the limit of detection (0.011 ng/mL), low exposure (0.011–10 ng/mL), and high exposure (≥10 ng/mL)^29^. The PIR was calculated by dividing household income by the poverty guidelines specific to the survey year, as also used in a previous study^30^. We also evaluated the PIR as a potential confounder in binary categories: low (<1), and high (≥1). BMI and waist circumference were evaluated in tertiles. Because physical activity is associated with CVRF, it was assessed by the amount of television, video game and computer usage daily and was added as a covariant in binary categories: high (≤2), and low (>2).

### Statistical methods

We regarded continuous variables as the mean ± standard deviation in the present study. The blood lead and CVRF variables underwent natural logarithmic transformation due to its skewed nature, and blood lead was included in the analyses as quartiles based on their distributions in the present population. To investigate the association between blood lead exposure and CVRF, we generated age-adjusted, gender-adjusted, and fully adjusted multiple variable linear regression models and present the associations with regression coefficients (Beta) and 95% confidence intervals (CIs). Multivariable linear models were used to assess the associations between interquartile ratio increases (IQ Ratio = 75th/25th percentiles of lead levels) in blood lead and LDL-C. Statistical tests for linear trends were conducted by modelling quartiles as an ordinal variable using integer values. We present the magnitudes of these associations as the average percent difference in CVRF within each IQ ratio group, as defined by the participants’ lead variables. These magnitudes were calculated as [(IQ Ratio^Beta) − 1] * 100. Alcohol consumption, waist circumference and diabetes were not included in the full adjusted models, as adjusting for these three variables separately or collectively did not change the results substantially (<5% of change in effect estimates). The Statistical Analysis Systems software package version 9.2 (SAS Institute, Inc.) was used for the analyses performed in this study. All P values are two-sided, and values less than 0.05 were considered to be statistically significant.

## Electronic supplementary material

Table S1

## References

[1] Kelly BB, Narula J, Fuster V (2012). Recognizing global burden of cardiovascular disease and related chronic diseases. The Mount Sinai journal of medicine, New York.

[2] Castellano JM, Narula J, Castillo J, Fuster V (2014). Promoting cardiovascular health worldwide: strategies, challenges, and opportunities. Rev Esp Cardiol (Engl Ed).

[3] Prendergast C, Gidding SS (2014). Cardiovascular risk in children and adolescents with type 2 diabetes mellitus. Current diabetes reports.

[4] Truong UT, Maahs DM, Daniels SR (2012). Cardiovascular disease in children and adolescents with diabetes: where are we, and where are we going. Diabetes technology & therapeutics.

[5] Roemmich JN, Lambiase MJ, Balantekin KN, Feda DM, Dorn J (2014). Stress, behavior, and biology: risk factors for cardiovascular diseases in youth. Exercise and sport sciences reviews.

[6] Vasconcellos F (2014). Physical activity in overweight and obese adolescents: systematic review of the effects on physical fitness components and cardiovascular risk factors. Sports Med.

[7] Ji F (2014). Genetic and environmental influences on cardiovascular disease risk factors: a study of Chinese twin children and adolescents. Twin research and human genetics: the official journal of the International Society for Twin Studies.

[8] Liu KS, Hao JH, Zeng Y, Dai FC, Gu PQ (2013). Neurotoxicity and biomarkers of lead exposure: a review. Chinese medical sciences journal = Chung-kuo i hsueh k’o hsueh tsa chih.

[9] Li MM (2014). The national trend of blood lead levels among Chinese children aged 0-18 years old, 1990–2012. Environment international.

[10] White BM, Bonilha HS, Ellis C (2016). Racial/Ethnic Differences in Childhood Blood Lead Levels Among Children <72 Months of Age in the United States: a Systematic Review of the Literature. Journal of racial and ethnic health disparities.

[11] Aoki Y (2016). Blood Lead and Other Metal Biomarkers as Risk Factors for Cardiovascular Disease Mortality. Medicine.

[12] Kaewboonchoo O (2010). Blood lead level and cardiovascular risk factors among bus drivers in Bangkok, Thailand. Industrial health.

[13] Kim KR, Lee SW, Paik NW, Choi K (2008). Low-level lead exposure among South Korean lead workers, and estimates of associated risk of cardiovascular diseases. Journal of occupational and environmental hygiene.

[14] Prokopowicz A (2017). Effect of occupational exposure to lead on new risk factors for cardiovascular diseases. Occupational and environmental medicine.

[15] Peters JL (2012). Lead concentrations in relation to multiple biomarkers of cardiovascular disease: the Normative Aging Study. Environmental health perspectives.

[16] Rhee SY (2013). Blood lead is significantly associated with metabolic syndrome in Korean adults: an analysis based on the Korea National Health and Nutrition Examination Survey (KNHANES), 2008. Cardiovascular diabetology.

[17] Ghiasvand M, Aghakhani K, Salimi A, Kumar R (2013). Ischemic heart disease risk factors in lead exposed workers: research study. J Occup Med Toxicol.

[18] Newairy AS, Abdou HM (2009). Protective role of flax lignans against lead acetate induced oxidative damage and hyperlipidemia in rats. Food and chemical toxicology: an international journal published for the British Industrial Biological Research Association.

[19] Abdel-Moneim AM (2015). Curcumin Ameliorates Lead (Pb(2+))-Induced Hemato-Biochemical Alterations and Renal Oxidative Damage in a Rat Model. Biological trace element research.

[20] Komousani TA, Moselhy SS (2011). Modulation of lead biohazards using a combination of epicatechin and lycopene in rats. Human & experimental toxicology.

[21] Herman DS, Geraldine M, T. V (2009). Influence of minerals on lead-induced alterations in liver function in rats exposed to long-term lead exposure. Journal of hazardous materials.

[22] Centers for Disease Control and Prevention. NCHS Research Ethics Review Board (ERB) Approval http://www.cdc.gov/nchs/nhanes/irba98.htm.

[23] Centers for Disease Control and Prevention; National Center for Health Statistics. National Health and Nutrition Examination Survey. Available at: https://wwwn.cdc.gov/nchs/nhanes/Default.aspx.

[24] Centers for Disease Control and Prevention; National Center for Health Statistics. National Health and Nutrition Examination Survey. Blood Metals Panel in whole blood. Laboratory Procedure Manual. Available at: https://www.cdc.gov/nchs/data/nhanes/nhanes_11_12/PbCd_G_met_blood%20metals.pdf.

[25] Centers for Disease Control and Prevention; National Center for Health Statistics. National Health and Nutrition Examination Survey.Total Cholesterol, HDL-Cholesterol, Triglycerides, and LDL-Cholesterol. Laboratory Procedure Manual. Available at: https://www.cdc.gov/nchs/data/nhanes/nhanes_03_04/l13_c_met_lipids.pdf.

[26] Centers for Disease Control and Prevention; National Center for Health Statistics. National Health and Nutrition Examination Survey. Plasma Glucose. Laboratory Procedure Manual. Available at: https://www.cdc.gov/nchs/data/nhanes/nhanes_03_04/l10am_c_met_glucose.pdf.

[27] Centers for Disease Control and Prevention; National Center for Health Statistics. National Health and Nutrition Examination Survey. Glycohemoglobin. Laboratory Procedure Manual. Available at: https://www.cdc.gov/nchs/data/nhanes/nhanes_07_08/ghb_e_met_tosoh_22_plus.pdf.

[28] Centers for Disease Control and Prevention; National Center for Health Statistics. National Health and Nutrition Examination Survey. Insulin. Laboratory Procedure Manual. Available at: https://www.cdc.gov/nchs/data/nhanes/nhanes_07_08/glu_e_met_insulin.pdf.

[29] Xu C, Liu Q, Zhang Q, Gu A, Jiang ZY (2015). Urinary enterolactone is associated with obesity and metabolic alteration in men in the US National Health and Nutrition Examination Survey 2001-10. The British journal of nutrition.

[30] Xu C (2015). Low Serum Testosterone Levels Are Associated with Elevated Urinary Mandelic Acid, and Strontium Levels in Adult Men According to the US 2011-2012 National Health and Nutrition Examination Survey. PloS one.

