# Supplementary material for: Associations between lead concentrations and cardiovascular risk factors in U.S. adolescents
Source: Sci Rep. 2017 Aug 22;7:9121. doi: 10.1038/s41598-017-09701-4 (PMC5567237; doi:10.1038/s41598-017-09701-4)
Supplement: Supplementary file 1 — Table S1 [file 41598_2017_9701_MOESM1_ESM.doc]

**Associations between lead concentrations and cardiovascular risk factors in U.S. adolescents**

Cheng Xu^1^,*, Yaqin Shu^1^,*, Zhi Fu^1,2^,*, Yuanli Hu^1^, Xuming Mo^1,#^

^1^ Department of Cardiothoracic Surgery, Children's Hospital of Nanjing Medical University, Nanjing, 210008, China.

^2^ Department of Thoracic Surgery, Huai'an First People's Hospital, Nanjing Medical University, Huai'an, 223001, China.

*****These authors contributed equally to the present study and should be regarded as joint first authors.

**^#^Correspondence to:**

Xuming Mo, Department of Cardiothoracic Surgery, Children's Hospital of Nanjing Medical University, 72 Guangzhou Road, Nanjing, 210008, China. Tel: 86-25- 83117234. Email: mohsuming15@sina.com

**Table S1. Mean and median of CVD risk factors and geometric mean and 95%CI by quartile of blood lead levels in US adolescents 1999–2012**

| CVD risk factors | All | All | Quartile 1 | Quartile 2 | Quartile 3 | Quartile 4 |
| --- | --- | --- | --- | --- | --- | --- |
|  | Mean | Median | Geometric mean (95% CI) | | | |
| Total cholesterol (mg/dL) | 160.62 | 157.00 | 158.85 (157.79, 159.92) | 157.37 (156.36, 158.39) | 158.17 (157.01, 159.34) | 157.04 (155.98, 158.11) |
| HDL-C (mg/dL) | 51.36 | 50.00 | 50.31 (49.88, 50.74) | 50.23 (49.81, 50.64) | 49.98 (49.53, 50.43) | 49.95 (48.81, 49.68) |
| LDL-C (mg/dL) | 90.95 | 88.00 | 87.17 (85.82, 88.54) | 87.01 (85.75, 88.30) | 86.58 (85.09, 88.09) | 88.13 (86.76, 89.51) |
| Fasting triglyceride (mg/dL) | 86.32 | 72.00 | 77.84 (75.85, 79.89) | 75.04 (73.09, 77.04) | 74.33 (72.37, 76.35) | 74.10 (72.28, 75.97) |
| Fasting glucose (mg/dL) | 93.03 | 92.00 | 92.05 (91.42, 92.69) | 92.24 (91.66, 92.82) | 92.91 (92.26, 93.57) | 91.92 (91.32, 92.52) |
| Glycohaemoglobin (%) | 5.19 | 5.20 | 5.18 (5.17, 5.20) | 5.17 (5.15, 5.18) | 5.18 (5.16, 5.20) | 5.16 (5.15, 5.18) |
| Fasting insulin (Uu/mL) | 14.18 | 10.85 | 12.04 (11.61, 12.49) | 11.33 (10.90, 11.76) | 11.10 (10.65, 11.57) | 10.40 (10.00, 10.81) |
| Systolic blood pressure (mmHg) | 109.75 | 109.00 | 108.47 (108.10, 108.84) | 109.32 (108.95, 109.69) | 109.71 (109.30, 110.13) | 109.71 (109.32, 110.09) |
| Diastolic blood pressure(mmHg) | 60.49 | 61.00 | 59.80 (59.34, 60.26) | 59.75 (59.28, 60.22) | 59.66 (59.15, 60.17) | 58.85 (58.35, 59.34) |

CVD, cardiovascular disease; HDL-C, high-density lipoprotein cholesterol; LDL-C, low-density lipoprotein cholesterol.

Lead(μg/dL), Quartile 1:<0.6; Quartile 2: 0.6-0.9; Quartile 3: 0.9-1.34; Quartile 4:>1.34.
